# A Self-Assembling Lipidic Peptide and Selective Partial V2 Receptor Agonist Inhibits Urine Production

**DOI:** 10.1038/s41598-020-64070-9

**Published:** 2020-04-29

**Authors:** Sunish Patel, Antonella Bavuso Volpe, Sahar Awwad, Andreas G. Schätzlein, Shozeb Haider, Boqian Liu, Ijeoma F. Uchegbu

**Affiliations:** 1 UCL School of Pharmacy, 29-39 Brunswick Square, London, WC1N 1AX UK; 2 Nanomerics Ltd. 30-34 New Bridge Street, London, EC4V 6BJ UK

**Keywords:** Cell biology, Drug discovery, Drug delivery, Pharmacology

## Abstract

Lipidised analgesic peptide prodrugs self-assemble into peptide nanofibers; with the nanofiber morphology protecting the peptide from plasma degradation and improving therapeutic efficacy. Extending this learning, we hypothesised that a self-assembling lipidized peptide arginine vasopressin (AVP) receptor agonist, that had not been designed as a prodrug, could prove pharmacologically active and control urine production. The only approved AVP receptor agonist, desmopressin is indicated for the treatment of central diabetes insipidus (DI), bedwetting, haemophilia A and von Willebrand disease. Desmopressin is well tolerated by most patients, however adverse effects, such as hyponatraemia and water intoxication necessitate a strict fluid intake, thus motivating the search for alternative DI treatments. Selective V2 receptor agonism is required for anti-DI activity and we hypothesised that our new lipidized peptide (METx) would lead to selective AVP receptor agonism. METx was synthesised and characterised and then tested for activity against the V2, V1a and OT uterine receptors and not tested against the V1b receptor as METx was not expected to cross the blood brain barrier. METx was also tested *in vivo* in a healthy rat model. METx forms nanofibers and is a partial V2 receptor agonist (determined by measuring MDCK cell line cAMP accumulation), producing 57% of AVP’s maximal activity (EC50 = 2.7 nM) and is not a V1a agonist up to a concentration of 1 μM (determined by measuring A7r5 cell line D-myo-inositol-1-phosphate accumulation). METx is a weak OT receptor antagonist, reducing the frequency of OT induced contractions (EC50 = 350 nM) and increasing the OT EC50 from 0.081 nM to 21 nM at a concentration of 600 nM. METx (41 nM) had no effect on spontaneous uterine contractions and METx (100 nM) had no effect on OT induced uterine contractions. Simulated binding studies show that binding avidity to the receptors follows the trend: V2 > OT > V1a. On intravenous injection, a nanoparticle formulation of METx reduced urine production in a healthy rat model in a dose responsive manner, with 40 mg kg^−1^ METx resulting in no urine production over 4 hours. The lipidized self-assembling peptide – METx - is a selective competitive V2 receptor agonist and an anti-diuretic.

## Introduction

Lipidized peptides self-assemble into nanofibers and such nanofibers have been used for tissue engineering and drug delivery^1–3^. In the case of drug delivery, the lipidized peptides are prodrugs as the active peptide epitopes are bound to a fatty acid chain via a cleavable ester linker^2,3^. The lipidized peptide prodrugs are more plasma stable and have a significant effect on pharmacological activity when injected via the intravenous route^3,4^. Previous work with fatty acid conjugated peptides had focused on analgesic peptide agonists of the opioid receptors (dalargin and leucine5-enkephalin)^3,4^ and in this work we sought to explore whether other receptor agonists could be prepared from self-assembling lipidized peptides. We hypothesised that an arginine vasopressin (AVP) agonist could be prepared from a lipidized peptide. The lipidized peptide has not been designed as a prodrug as the lipid moiety is conjugated to the peptide epitope via an amide bond instead of an ester bond^2,3^, as had been reported previously.

The vasopressor effect of arginine vasopressin (AVP) was first reported in 1895 after discovering that the extracts of cow pituitary had a vasopressor effect^5^. This was followed by the work of Du Vigneaud in the 1950s, who discovered the structure of AVP and oxytocin (OT) and synthesised these compounds^6,7^. Since then, both AVP and OT have been the focus of intensive research as both agonists and antagonists have various therapeutic uses and are also used as research tools^8^. AVP is active at three known receptors: V1a receptors in the smooth muscle and liver resulting in pressor activity, V1b receptors largely found in the pituitary gland resulting in adrenocorticotrophic hormone releasing activity and V2 receptors found in the kidney collecting ducts that result in anti-diuretic activity^8^.

**Figure 1:**
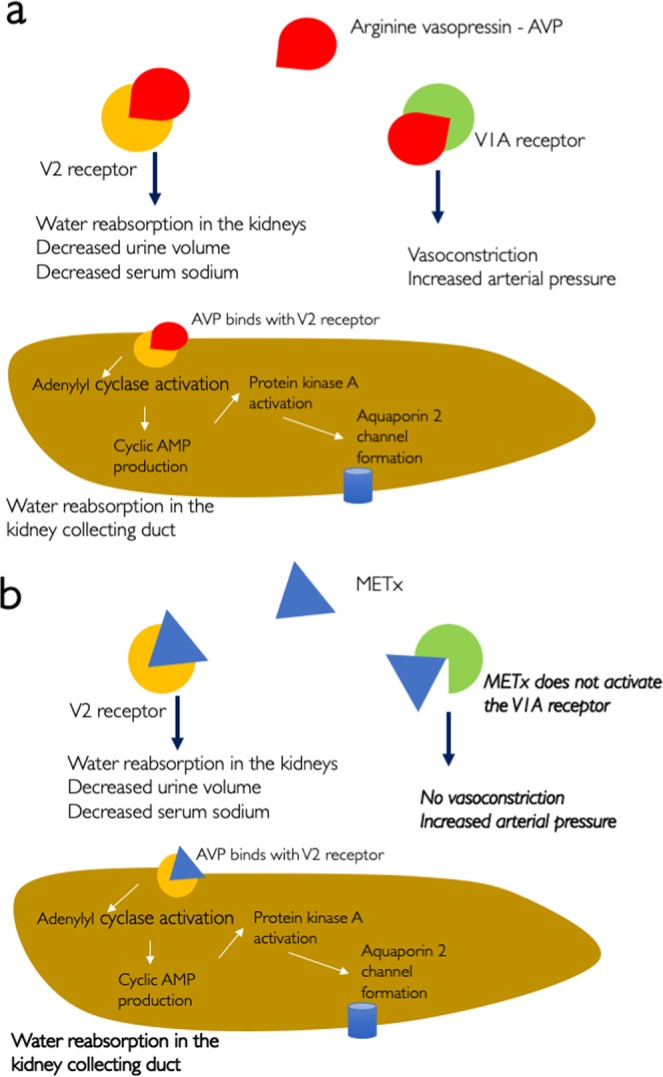
The activity of: (**a**) AVP and (**b**) METx, at the V2 and V1a receptors. METx antagonist activity at the OT receptor has also been observed at a concentration that is 100 fold higher than the V2 receptor EC50.

AVP agonists and antagonists may be used to treat a variety of conditions. The most common use of an AVP agonist, is the use of desmopressin for the treatment of central diabetes insipidus /

(DI)^9^, where the V2 receptor is targeted. Central, or neurohypophyseal, DI is caused by a lack of AVP^10^. AVP acts by stimulating adenyl cyclase to produce cAMP which activates Protein Kinase A, causing the phosphorylation and translocation of aquaporin-2 to the membrane and thus enabling water reabsorption in the kidney collecting duct^11,12^ (Fig. 1). AVP is normally produced by the hypothalamus and stored and secreted by the neurohypophysis^10^. The lack of AVP or its activity results in excessive thirst (polydipsia) and the production of large volumes of urine (polyuria)^10^. Polyuria can present as increased urinary frequency, incontinence and/ or nocturnal enuresis. Desmopressin is also approved for the treatment of nocturnal enuresis^13^. Desmopressin is well tolerated by the majority of patients, however the risk of adverse effects, such as hyponatraemia and water intoxication require a strict fluid intake with little room for variability^14^.

**Figure 2:**
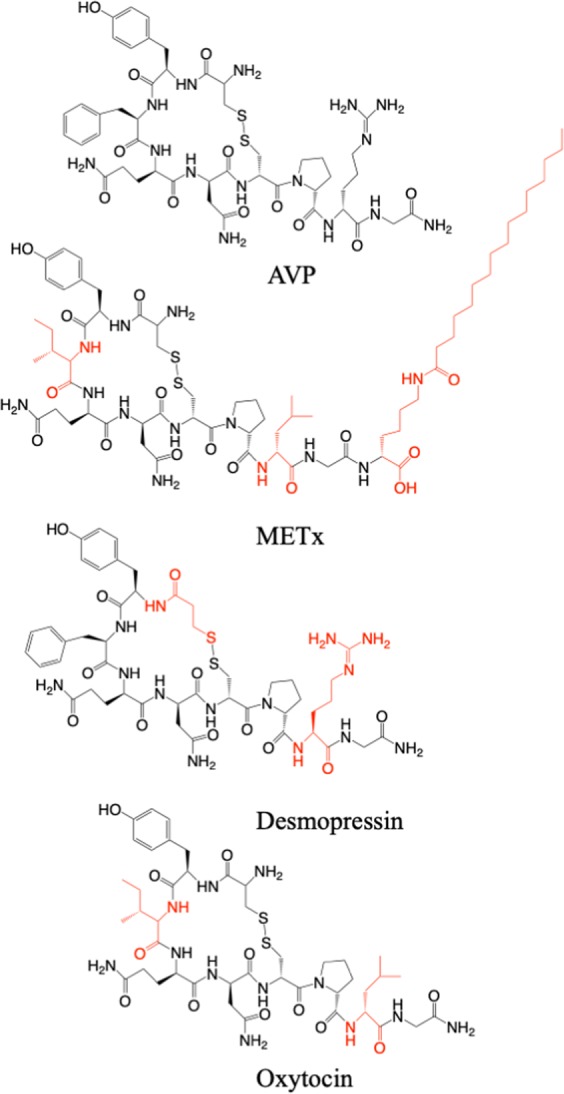
Chemical structures of AVP, METx, desmopressin and oxytocin (OT).

Structurally, desmopressin differs from AVP in two regions (Fig. 2): deamination of the cysteine residue at position 1 and substitution of L-Arginine with D-Arginine at position 8. Structure activity studies have revealed the 20 member cyclic ring to be essential for both pressor and oxytocic activity^15^ and that the carboxy terminus was essential for activity at the AVP receptors^16^.

Efforts to develop non-peptide agonists and antagonists of AVP and OT have resulted in the approval of only two non-peptide antagonists of the AVP receptors: tolvaptan, a V2 antagonist and conivaptan, a V2/ V1a antagonist; with many large relevant drug discovery programmes abandoned by pharmaceutical companies^8^. Selective peptide agonists and antagonists should thus be explored as potential therapeutics, as they offer several advantages over small molecule non-peptide analogues, in that they do not result in the build-up of toxic metabolites and are usually highly specific for the receptor family in question^17^. However, receptor selectivity is a challenge and selectivity in rodents does not automatically confirm that selectivity will be obtained in humans^8^. For example, desmopressin does not show any affinity for the human V1a receptor^8^ but is a potent agonist of the human V1b receptor with a lower EC50 agonist activity than against the human V2 receptor^18^.

We sought to examine if a lipidized peptide, which assembles into nanofibers could serve as a selective V2 receptor agonist and control urine production. Such a compound may serve as an alternative to desmopressin, although a desmopressin replacement is not the focus of this work. We thus designed METx (Fig. 2). METx differs from AVP by having isoleucine replacing phenyl alanine at position 3, leucine replacing arginine at position 8 and a palmitoyl lysine to the end of position 9 (Fig. 2). METx is a hydrophobic lipidized peptide and closely resembles OT, essentially having a palmitoyl lysine unit added to position 9 on OT (Fig. 2). Here we report on the activity of METx at the uterine OT, V1a and V2 receptors, as well as its effects on urine production.

## Materials and Methods

### Materials

All chemicals were obtained from Sigma Aldrich unless otherwise indicated. METx (Fig. 2) was designed in house and custom synthesised by Thermo Fisher Scientific, UK. Nanomerics’ Molecular Envelope Technology (N-palmitoyl-N-monomethyl-N,N-dimethyl-N,N,N-trimethyl-6-O-glycolchitosan, GCPQ, Mw = 7.12 kDa, Mw/ Mn = 1.004, Mole% palmitoylation = 25%, Mole% quaternary ammonium groups = 12%) was obtained from Nanomerics Ltd. TrypLE™ Express was obtained from Thermo Fisher Scientific, UK. The IP-One ELISA kit was obtained from Cisbio Bioassays, USA and the cAMP Biotrak enzyme immunoassay kit was obtained from GE Life Sciences, USA. A7r5 rat smooth muscle cells and Madin-Darby Canine kidney (MDCK) cells were obtained from European Collection of Cell Cultures (ECACC). Male Wistar rats were obtained from Charles River (Charles River, UK) while Male Sprague Dawley rats were obtained from Harlan (Harlan, UK).

### Physical Characterisation of METX

The critical micellar concentration (CMC) of METx was determined using the pyrene assay^19^. An aqueous solution of pyrene (2μM) was prepared by dissolving pyrene in absolute ethanol (0.4 mg ml^−1^). Under a stream of nitrogen, the pyrene solution (100μl) was dried in a 100 mL volumetric flask. The solution was made up to 100 mL using MilliQ water. Using this pyrene solution as the solvent, various concentrations of METx were prepared 2 pg ml^−1^ − 200 μg ml−^1^ (1.45 × 10^−10^ to 1.45 × 10^−4^M). Each solution was probe sonicated on ice for 15 minutes with a cycle of 5 minutes on and 3 minutes off with a Q Sonica probe sonicator (Cole Parmer Instruments Co. Ltd, UK). The Q Sonica was used with a microtip and set to 25% of its maximum output. The fluorescence spectra was recorded using a spectrophotometer (LS 50B Fluorescence Spectrometer, Perkin Elmer, Massachusetts, United States). The excitation was set at 335 nm and the emission was measured from 350 to 500 nm. The ratios of the intensity of the third (383 nm) and first (375 nm) peaks in the emission spectra (the I3/I1 ratio) were recorded and plotted against concentration. The CMC was determined as the concentration at which a sharp change in the gradient of the curve is observed. Each concentration was prepared and measured in triplicate.

### Receptor Activity Studies

There is good correlation in the agonist binding activity on OT rat and human receptors^20^ and good correlation between the agonist binding activity on V1a human and rat receptors^21^. Finally the dog and human V2 binding kinetics of radiolabelled AVP are comparable^22^. We thus used the V2 receptor from MDCK cells, V1a receptors from A7r5 cells and OT receptors from rat uterine strips for our receptor activity assays.

#### V2 receptor

MDCK cells expressing the V2 receptor were cultured in Eagle’s Essential Minimum Medium (EMEM) supplemented with foetal bovine serum (FBS, 10% v/v), L-Glutamine (1% v/v) and non-essential amino acids (NEAA 1% v/v). Cells were grown at 37 °C in T-75 cm^2^ culture flasks under a humidified (95% air/ 5% CO_2_) atmosphere. The cell culture medium was changed every 2 days. Cells were sub-cultured every 7 days or upon reaching confluence using trypsin/ ethylene diamine tetraacetic acid (EDTA) (0.25%w/v trypsin/ 0.01%w/v EDTA in phosphate buffered saline, 3 mL) solution to dissociate the cells and the cells reseeded at one third of their original density.

To determine the dose response curve, MDCK cells (passage number 10–20) were detached as described above using trypsin and seeded in transwell plates (12 mm transwell with 0.4μm pores) at a concentration of 500,000 cells per transwell and grown for 72 hours. The cell culture media was changed daily. On the day of the experiment, the cells were washed with Dulbecco’s phosphate-buffered saline (DPBS) without calcium and magnesium and incubated for 1 hour in cell culture medium with isobutyl methylxanthine (IBMX, 0.5 mM). IBMX is a phosphodiesterase inhibitor that prevents the breakdown of cAMP within the cells; allowing for total quantification of the analyte cAMP. After this, cells were incubated for 30 minutes with various concentrations of AVP or METx in cell culture medium and in the presence of IBMX (0.5 mM). In order to reduce the concentration gradient of AVP or METx from the apical to the basolateral regions and vice versa, cells were treated from both the apical and basolateral chambers with the given concentration of AVP or METx dissolved in Dulbecco’s phosphate buffered saline (DPBS) free of calcium or magnesium ions and with IBMX (0.5 mM, 0.5 mL in the transwell insert and 1.5 mL in the plate).

After 30 min incubation with the respective concentration of AVP or METx, the cells were lysed using the cAMP enzyme immunoassay’s (EIA’s) lysis buffer (diluted lysis reagent 1B, 0.5 mL, dodecyltrimethylammonium bromide 0.25% w/v, bovine serum albumin 0.02%w/v, preservative 0.01%w/v, acetate buffer 0.05 M, pH = 5.8) and agitated for 10 minutes. cAMP was quantified from the cell lysate (0.1 mL) using a competition based EIA as detailed in the Supplementary Information.

##### Competition Studies

The same experimental set up as described above was followed for the seeding of MDCK cells and incubation with IBMX (0.5 mM) on the day of the experiment. For competition studies, the cells were incubated with a high concentration of AVP (10^−7^M) and varied concentrations of METx (10^−13^ to 10^−7^M). After 30 minutes of incubation with the respective concentration of AVP and METx, the cells were lysed using the cAMP EIA’s lysis buffer for 10 minutes as per the cAMP EIA protocol outlined above.

#### V1a receptor

A7r5 cells were cultured in Dulbecco’s Modified Eagle’s Medium (DMEM) with high glucose concentrations (4.5 g/L glucose) and foetal bovine serum (FBS, 10% v/v). Cells were grown at 37 °C and under a humidified 95% air /5% CO2 atmosphere in T75 cm^2^ or T150 cm^2^ culture flasks. The cell culture medium was changed every 2 to 3 days. When cells reached sub-confluence (80% to 90% coverage), they were subcultured using TrypLE™ Express (TrypLE™ Express is a replacement of the traditional trypsin reagent) and the cells reseeded at one third of their original density.

The IP-1 ELISA was carried out using the IP-One ELISA kit. A7r5 cells (passage number: 20–23) were washed once in Dulbecco’s Phosphate Buffered Saline (DPBS) and detached as described above using trypsin and then seeded in a 96 well plate at a concentration of 60,000 cells per well (in a 200 μL volume). The cells were incubated as described above for 22 hours. On the day of the experiment, the cell supernatant was then removed. The cells were then incubated with various concentrations of METx, AVP or desmopressin dissolved in the IP-One Stimulation Buffer (Hepes 10 mM, CaCl_2_ 1 mM, MgCl_2_ 0.5 mM, KCl 4.2 mM, NaCl 146 mM, Glucose 5.5 mM, LiCl 50 mM, pH = 7.4, 30 μL), with a set of control wells receiving only the IP-One Stimulation Buffer. After 1 hour of incubation with the respective concentrations of METx, AVP or desmopressin, the cells were then lysed using the IP-One Lysis Reagent (30 μL) by incubation as described above for a further 30 minutes. An aliquot of the cell supernatant (50 μL) was placed in the ELISA plate and the cell lysates analysed as described in the Supplementary Information.

#### OT receptor

##### Preparation of uterine strips

Female Wistar rats (203–225 g) were killed with CO_2_ on the day of the experiments. The uterus was excised and dissected into longitudinal strips (0.5 ×1.3–1.4 cm) from the anti-mesometrial side of the mid-portion of horns which contained implantation sites.

##### Contraction studies

The uterine strips were suspended in an organ bath that contained physiological salt solution (PSS, 25 ml: 116 mM NaCl, 4.6 mM KCl, 1.16 mM NaH_2_PO_4_.H2O, 1.16 mM MgSO_4_.7H2O, 21.9 mM NaHCO_3_, 1.8 mM CaCl_2_.2H2O, 11.6 mM dextrose, 0.03 mM CaNa_2_EDTA, pH = 7.5 ± 1). The water-jacketed bath was maintained at 36 °C and aerated with a mixture of 95% O_2_ and 5% CO_2_. Only one strip from each animal was used. The uterine strip was tied at the bottom end to a fixed metal hook and at the top end to a force displacement transducer (Harvard Instruments, Kent, UK) using a cotton thread [17]. The force displacement transducer was coupled to an amplifier driving a direct writing oscillograph (Universal Oscillograph, Harvard, Kent, UK). A cumulative dose procedure was used and isotonic contractions recorded.

To quantify the effect of METx on uterine OT induced contractions the myometrial strips were equilibrated for 60 min within the organ bath and then a single dose of OT applied (17 µg mL^−1^, 2 µL to give an extracellular concentration of 1.35 nM OT) followed by a single dose of DMF and doses of METx in PSS (diluted originally from a stock solution in dimethylformamide - DMF) as indicated in Table 1 (Supplementary Information). DMF was added as DMF had been used to solubilise METx. DMF had no effect on the measured uterine contractions (Fig. S1, Supplementary Information). The mean peak amplitude (MPA) was calculated from Eq. 1.

1 $${\rm{MPA}}=\frac{\varSigma PH}{n}$$

where PH = peak heights obtained within the 10 minute period immediately after addition of METx, OT or dimethylformamide and n = number of peaks within the same 10 minute period. The frequency of isotonic contractions was also evaluated during the 10 minute period immediately after the addition of METx, OT or dimethylformamide. The concentration of METx in the organ bath ranged from 0.045–0.586 nM.

**Table 1:** Double reciprocal plots of response vs OT concentration (1/ R vs 1/[OT]).

Inhibitor	Concentration (nM)	Linear equation	r^2^	1/Rmax
None		$\frac{1}{R}=4.01\times {10}^{11}\frac{1}{[OT]}+0.45$	0.960	0.45
Atosiban	100	$\frac{1}{R}=6.21\times {10}^{10}\frac{1}{[OT]}+0.47$	0.96	0.47
METx	100	$\frac{1}{R}=6.12\times {10}^{11}\frac{1}{[OT]}+0.48$	0.99	0.48
METx	300	$\frac{1}{R}=9.33\times {10}^{11}\frac{1}{[OT]}+0.97$	0.97	0.97
METx	600	$\frac{1}{R}={10}^{-9}\frac{1}{[OT]}+1.33$	0.99	1.33

R = contractile response in cm, Rmax = maximum contractile response in cm, [OT] = OT concentration in moles per litre.

To quantify the effect of METx on spontaneous uterine contractions the myometrial strips from the same uterus as above, but from the opposite horn, were equilibrated for 60 min within the organ bath and then a single dose of DMF applied followed by doses of METx as shown in Table 1 in the Supplementary Information. The contraction frequency during the 10 min segment after 60 mins equilibration was termed the basal frequency.

To study the effect of METx on the OT dose response curve, after the uterine strips had been equilibrated for a 60 min period, strips were challenged with KCl (3.06 M, 0.5 mL, final concentration in bath = 60 mM) to determine maximum KCl-induced contraction force. After rinsing out the KCl, dimethylformamide (100 µL) was added followed by increasing doses of OT (starting at a dose of 0.17 µg mL^−1^, 6μL and doubling the dose every 5 minutes) to obtain a dose response curve at an OT concentration of (0.0405–86.4 nM). The organ bath was not washed out until a maximal response was reached. The MPA was calculated as shown in Equation 1, except that the recordings were done after the addition of each dose of OT and the MPA was calculated over a 5 minute period. To quantify the effect of METx on OT induced contractions, METx (final bath concentration = 0.1, 0.3 or 0.6 µM) was added to the organ bath prior to the addition of OT (Table 2 – Supplementary Information). To compare the nature of the METx antagonism to a known OT receptor antagonist, atosiban^23^ was included in this assay. Atosiban is approved for use as an anti-abortion drug^23^. Results were plotted as a percentage of the maximum contraction obtained with KCl against concentration. A double reciprocal plot was used to assess the nature of the receptor antagonism observed.

### In Silico Receptor Binding

There is no crystal structure present for rat V1a, V2 or OT receptors in the Protein Data Bank. The structure of the closest homolog that can used as a template to study V1a receptor is the human δ-opioid receptor (PDB ID 4RWA), with an overall sequence identity of 30%. This identity is ~72% around the bifunctional peptide-binding site, thus permitting the construction of an accurate homology model. Although there is a higher resolution structure present (PDB ID 4N6H), the 4RWA structure was chosen because it is in a complex with a bifunctional peptide and provides an excellent basis to understand cyclic METx peptide binding to the receptors. The sequences for the rat receptors were downloaded from UniProt (V1a: id P30560; V2: Q00788 and OT: P70536) and aligned using Clustal Omega^24,25^ The sequence alignment indicated that the regions that can be modelled accurately are residues 42–371 (V1a), 32–341 (V2) and 34–345 (OT). Homology modelling was carried out in two steps. In the first step, a model of V1a receptor was built. In the second step, the model of V1a receptor was used as a template to model V2 and OT receptors. The overall sequence identity between V1a and V2 receptor is 46% over 309 residues and between V1a and OT receptor is 56% over 311 residues. The models were built using Internal Coordinate Mechanics method implemented in the Molsoft ICM software^26^ and stereochemical parameters checked using PROCHECK^27^. In all cases the final model was chosen based on the low-energy function and low Cα rmsd between the template and the model. Several rounds of minimisation were performed to relieve steric clashes between side chains. The final Cα rmsd between 4RWA and V1a receptor was 0.17 Å; V1a and V2 receptor was 0.13 Å and V1a and OT receptor was 0.12 Å. The models were structurally superimposed on 4RWA, to identify the corresponding analogous residues that formed a part of the bifunctional peptide binding site. This was based on the assumption that the peptide would interact in the similar manner and replicate all conserved interactions from the template into the model. These residues were then used to dock the METx cyclic peptide head group. The peptide part was sketched using LigEdit module and docked using the method implemented in the ICM-Pro modelling suite using default values^26^. In each case, the best dock was chosen in which the hydrocarbon tail could be extended from the cyclic METx peptide and did not interfere with the binding of METx to the receptor. Finally, the hydrocarbon tail was minimised to relieve any steric clash with the receptor. Molecular graphics images were generated using the ICM browser^11^. We carried out this docking, based on our previous experience of working with lipids interacting with proteins in a membrane environment^28^ More specifically it would be thermodynamically unfavourable for a charged cyclic peptide to sit in a hydrophobic membrane environment. Therefore the plausible model based on other available crystal structures is that the cyclic peptide would be interacting within the receptor and the hydrophobic tail of METx would be anchoring the cyclic peptide to the hydrophobic environment. On a more technical note, the number of rotatable bonds in the hydrophobic tail, combined with the number of conformations that one bond can adopt makes the docking exercise almost impossible with the lipid. Therefore a conformation of the peptide is chosen on which a lipid tail can be added and extended into a membrane. This is followed by a round of minimisation and restrained dynamics to relieve any steric clashes between the lipid tail and the protein.

### METx Nanoparticle Formulations

#### Preparation of nanoparticles

METx nanoparticles were prepared using GCPQ, a self-assembling polymer which forms drug loaded nanoparticles and which protects peptides from degradation in the plasma^2,3,29–31^. GCPQ polymer nanoparticles were thus used to protect METx from plasma degradation. On intravenous injection GCPQ from the nanoparticles is completely excreted in the urine within 24 hours^2^. A mixture of the GCPQ (5 mg) and METx (1 mg), dispersed in water was vortexed followed by probe sonication using a QSonica Sonicator with a micro tip (Cole Parmer Instruments Co. Ltd, UK) for 15 minutes on ice, with a cycle of 5 minutes on and 3 minutes off for cooling. The instrument was set at 30% of its maximum output.

The resulting drug loaded nanoparticles were then imaged using transmission electron microscopy (TEM)^3^. TEM was performed by placement of a drop of the nanoparticle formulation on a Formvar/carbon coated grid. Excess sample was blotted off with Whatman No. 1 filter paper and the sample was negatively stained with uranyl acetate (1% w/v). Samples were imaged using a CM120 BioTwin Transmission Electron Microscope (Philips, USA). Images were captured using an AMT digital camera (Woburn, USA).

Particle sizing was carried out using a Malvern Nanosizer (Malvern Instruments, UK) at 25 °C, a wavelength of 633 nm and a detection angle of 90°. Data was analysed using the Contin method of analysis.

#### Plasma stability

The plasma stability of METx was evaluated prior to an *in vivo* study. Blood was collected from male Sprague Dawley rats (n = 3, 200–250 g) into a vacutainer coated with ethylene diamine tetra-acetic acid (EDTA) and kept on ice (4 °C) until the plasma could be separated by centrifugation (10 min at 2000g and 4 °C, Hermle z323k centrifuge, HERMLE Labortechnik, Germany). The separated rat plasma was stored at −20 °C until required. On the day of the experiment, the plasma was thawed just prior to the experiment and diluted to 30% v/v using NaCl (0.9%). METx (7.2 × 10^−4^M, 50 μL) was added to the diluted plasma (30% v/v, 450μL) that had been allowed to warm for 30 minutes at 37 °C either as a solution in dimethylsulfoxide or as a GCPQ nanoparticle formulation dispersed in water. The formulations and free drug were incubated at 37 °C with shaking for a maximum of 4 hours. At regular time intervals aliquots (50μL) were removed and mixed with dimethylformamide (150μL) to dissolve the METx and precipitate the plasma proteins. Samples were then immediately vortexed and centrifuged for 10 minutes at 13,000 rpm (MSE micro centaur centrifuge, MSE, UK). The supernatant (100μL) was collected and 40 μL was analysed using a reverse phase high performance liquid chromatography (HPLC) method. HPLC analyses was performed using an Agilent 1200 HPLC system (Agilent Technologies, UK) equipped with a quaternary pump, degasser, autosampler and a UV detector. Samples were chromatographed over a reverse phase column: Phenomenex Onyx Monolithic C18 column (5μm, 4.6 ×100 mm, Phenomenex, UK) connected in series with a guard column (5μm, 4.6 ×10 mm). The column temperature was kept constant at 35 °C. Separation was performed using an acetonitrile, water mobile phase gradient method (Table 3 – Supplementary Information) at a flow rate of 1.5 mL per minute, with detection at a wavelength of 280 nm. The chromatograms were analysed using HPLC Chemstation (version 01.05 for windows, Agilent Technologies, UK) software.

### Animal Studies

Within the UK, animal experiments are regulated by a law known as the Animal Scientific Procedures Act 1986. All animal studies are conducted under a licensing system. In the current study all the experiments were performed under a Home Office License No. PPL 70/8224 (Animals Scientific Procedures Act 1986) and all procedures were approved by the local Animal Scientific Procedures Ethics Committee of University College London and all procedures were carried out in accordance with the relevant guidelines and regulations.

Male Sprague Dawley rats (220–250 g) were housed five per cage in an air conditioned unit maintained at 20–22 °C and 50–60% humidity, with lighting controlled on a twelve-hour cycle. The lights were switched on at 07:00 and off at 19:00. Animals were allowed free access to standard rodent chow and water. Before experimentation, animals were habituated for 7 days within the unit. Prior to testing animals were acclimatised to the procedure room for 1 hour. Animals were also acclimatised to the metabolic cages for 20 minutes per day for 5 days before the day of experiment. Animals were randomly assigned into one of four groups; untreated, and animals receiving an intravenous injection of 10, 20 or 40 mg kg^−1^ of METx as a GCPQ, METx (5: 1) nanoparticle formulation (n = 3) in a dose volume of 0.9 mL. The formulations were freshly prepared on the morning of the experiment as described above and the pH of the formulation adjusted to pH = 7 using sodium hydroxide (1 M). After receiving their METx formulations, animals were placed within individual metabolic cages (Techniplast, Italy) with access to water. Spontaneously voided urine was collected for 4 hours. Urine osmolality was determined by freezing point depression using a Type 5r osmometer (Loser, Germany).

### Statistical Analyses

All the results were expressed as a mean ± standard deviation (SD). Statistical analyses was carried out using the one-way ANOVA test when more than 2 groups were compared with post-hoc analysis (Tukey’s Test) to determine statistical significance. Statistical significance was set at a p value of less than 0.05.

## Results

### METx Physical Characterisation

**Figure 3:**
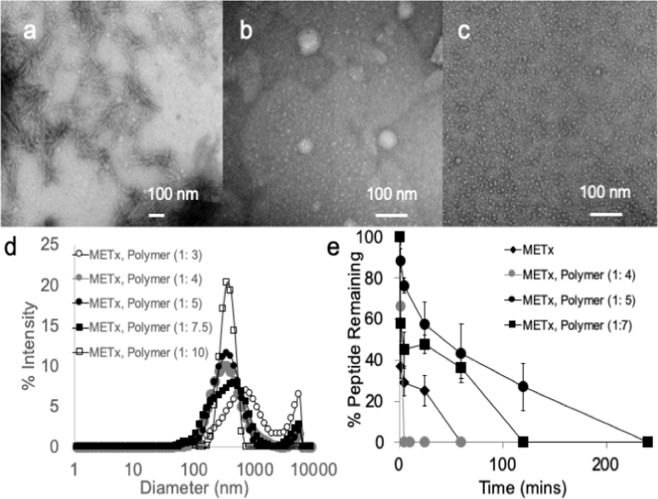
METx physical characterisation: negatively stained transmission electron micrograph (TEM) of (**a**) METx 1 mg mL^−1^ and (**b**) METx polymer nanoparticles (METx: polymer weight ratio = 1: 5, METx concentration = 1 mg mL^−1^) in water, (**c**) METx polymer nanoparticles (METx: polymer weight ratio = 1: 10, METx concentration = 1 mg mL^−1^) in water. (**d**) Size distribution graphs (intensity) of the various METx, polymer nanoparticle formulations, (**e**) METx (final concentration = 0.1 mg mL^−1^) stability when incubated with 30% rat plasma at 37 °C (mean ± s.d.). For the plasma stability studies, the METx, Polymer (1: 5) formulation is significantly different from the METx alone, METx, polymer (1: 4) and METx, Polymer (1: 7) formulations (p < 0.05) and the METx, Polymer (1: 7) formulation is significantly different from the METx alone and METx, Polymer (1: 4) formulations (p < 0.05). Data is representative of at least two independent experiments.

We studied the self-assembly of METx in aqueous media in order to explore if nanofibers would be formed from the lipidized peptide, as lipidized peptides are known to self-assemble into nanofibers^2–4^. METx spontaneously self assembles into nanofiber structures in aqueous media with a diameter of about 20 nm and lengths of 200–500 nm (Fig. 3a). The critical aggregation concentration of METx in water is 24.7 mg L^−1^ (18 μM). The self-assembly is driven by the aggregation of the palmitoyl chains, which in turn causes an entropy gain in the water molecules bordering the hydrophobic cavity^32^. Peptide nanofibers form from lipidized peptides containing beta-sheet forming amino acids (aromatic amino acids, isoleucine and valine) with the beta sheet formation (facilitated by inter-molecular hydrogen bonding), preventing the self-assembly into spheres and instead enabling nanofiber self-assemblies^4,33^. A spherical self-assembly is normally enabled by the repulsion between the hydrophilic head groups of amphiphilic molecules^34^. The molecular arrangement of peptide nanofibers has been described previously and nanofiber self-assemblies usually protect the peptide part of the molecule from plasma degradation^4,33^. In the presence of GCPQ, a polymer known to protect peptides from plasma degradation^2^, the nanofibers transformed to spheres (Fig. 3b–d) and the polymer protected the peptide from plasma degradation (Fig. 3d). Having established the fact that self-assembling peptide nanofibers are formed from METx, we then set about testing the *in vitro* functional activity of METx at the V2, V1a and OT receptors.

**Figure 4:**
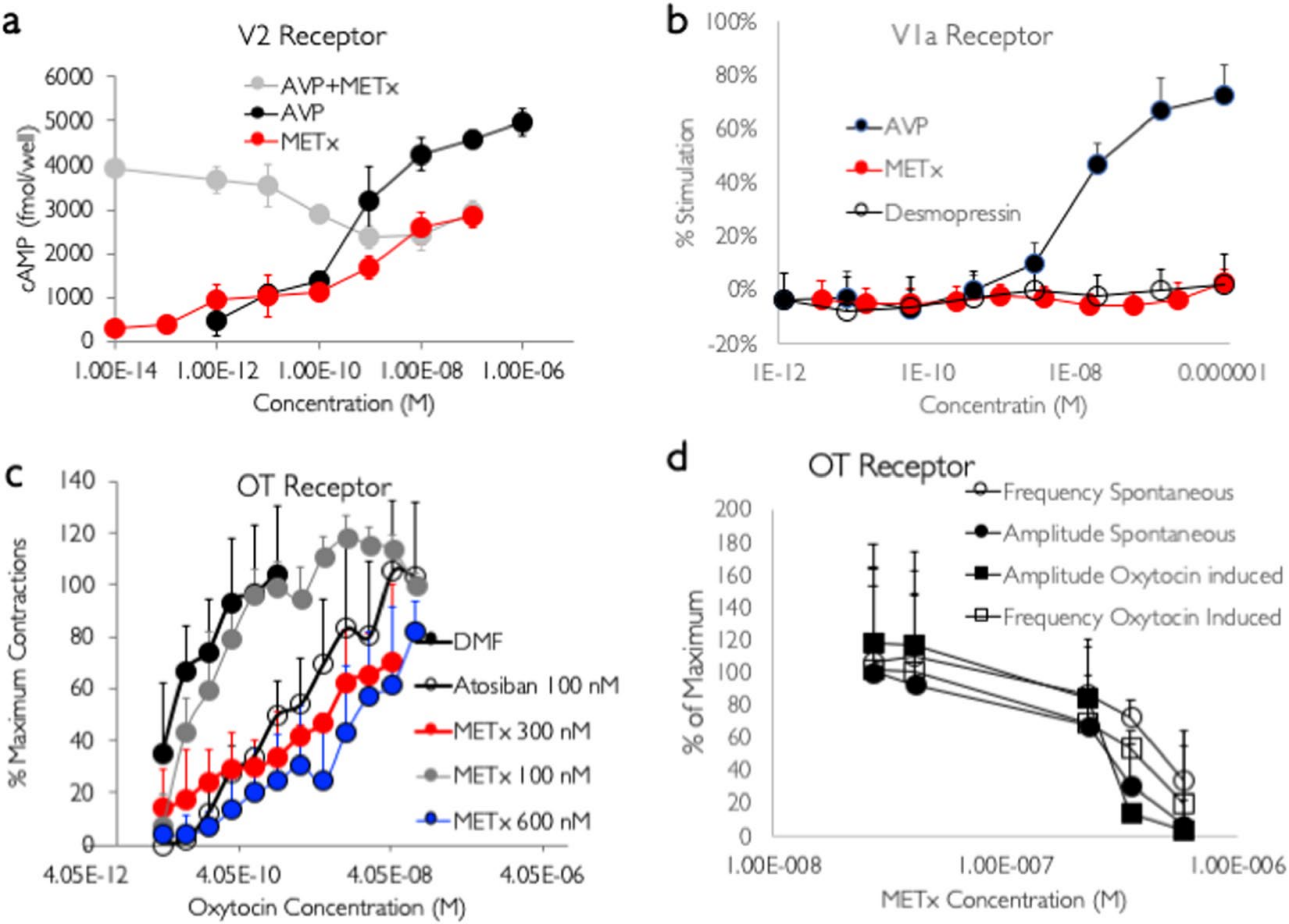
METx (mean ± s.d.) activity at the: (**a**) V2, (**b**) V1a, (**c**) OT receptor (OT induced contractions) and (**d**) OT receptor (spontaneous and OT induced contractions at an OT concentration of 1.35 nM). Representative assay data from at least two independent experiments.

### Receptor Binding

MDCK cells were seeded in transwell plates as 75% of the V2 receptors are expressed on the basolateral membrane^35^. This allowed maximal access to the V2 receptors. AVP and METx produced a dose dependent increase in cAMP production (Fig. 4a). The maximal response for AVP was seen at 100 nM and the AVP EC50 was 0.93 nM (Fig. 4a). METx also produced a dose dependent increase in cAMP, however its maximal response was seen at 10 nM and the maximum cAMP response for METx was 57% of that seen with AVP and as such METx showed partial agonist activity of the V2 receptor. METx had an EC50 of 2.7 nM (Fig. 4a). On application of both METx and AVP to the MDCK cells there was a gradual inhibition of AVP agonism until the activity reached the Emax of METx (Fig. 4a), indicating that METx is actually an antagonist of AVP at the V2 receptor as well as having its own agonist activity. With the application of both METx and AVP, the decrease in cAMP production started to take place at a similar concentration to the EC_50_ of METx (Fig. 4a). This suggests that METx binds to the same site as AVP at the V_2_ receptors of MDCK cell lines. The results also suggest that METx has a higher affinity for the V_2_ receptor than AVP as cAMP production shifts to a similar level to that produced by METx when both METx and AVP are applied to the MDCK cells. As the main focus of this paper is an exploration of the pharmacological activity at certain receptors of a lipidized peptide (METx), desmopressin, an approved V2 receptor agonist indicated for the treatment of diabetes insipidus and nocturnal enuresis^9^, was not employed in this assay as it is known that the functional activities (fluid secretion) of AVP and desmopressin in the MDCK cell line are comparable with EC50 values of 0.36 and 0.7 nM respectively^36^. METx was not an agonist to the V1a receptor in the A7r5 rat smooth muscle cell line, up to a concentration of 1 μM and there was a good V1a response to AVP in this assay, as AVP had an EC50 of 13.1 nM (Fig. 4b). Additionally, desmopressin, an approved V2 receptor agonist indicated for the treatment of diabetes insipidus and nocturnal enuresis^9^, was not an agonist at the V1a receptor (Fig. 4b).

However, METx was found to be an antagonist at the OT receptor; reducing the amplitude of OT induced contractions, in isolated uterine strips, at METx concentrations of 300 nM and 600 nM; shifting the dose response curve to the right by two orders of magnitude (Fig. 4c, p < 0.05). An OT concentration of 1.35 nM induced enhanced contractile activity in the uterine strips and these were termed as OT induced contractions. The maximum effect of OT was reduced by about 20% in the presence of METx and higher levels of OT could not induce the same magnitude of contractions in the uterine strips, an indication that METx is a non-competitive antagonist at the OT receptor. METx was not as effective as atosiban, an approved anti-abortion medication^23^, at reducing OT induced contractions as 100 nm atosiban shifted the OT induced contraction-concentration curve to the right (Fig. 4c, p < 0.05), whereas 100 nm of METx was not effective in reducing OT induced contractions (Fig. 4c, p > 0.05). METx reduced the frequency and amplitude of spontaneous uterine contractions at a METx concentration of 350 nM (Fig. 4d).

While atosiban has a similar 1/Rmax to OT (Table 1), the effective concentrations of METx (300 and 600 nM) have 1/ Rmax values in excess of that observed with OT; indicating that while atosiban is a competitive antagonist of the OT receptor, METx is a non-competitive antagonist of the OT receptor.

METx produces a maximal partial agonist response at the V2 receptor at 10 nM, is inactive at the V1a receptor up to a concentration of 1 μM and is active as an antagonist at the OT receptor starting at a concentration of 300 nM (Fig. 4). Tests on the V1b receptor were not carried out as it was deemed unlikely that METx would cross the blood brain barrier in sufficient quantity due to its rapid degradation in plasma (Fig. 3e). The V1b receptors reside mainly. In the anterior pituitary gland^37^

METx a lipidized, nanofiber forming peptide and AVP analogue (Figs. 2 and 3) is active at the OT and V2 receptors but not active at the V1a receptor (Fig. 4).

### METx In silico Receptor Binding Studies

**Figure 5:**
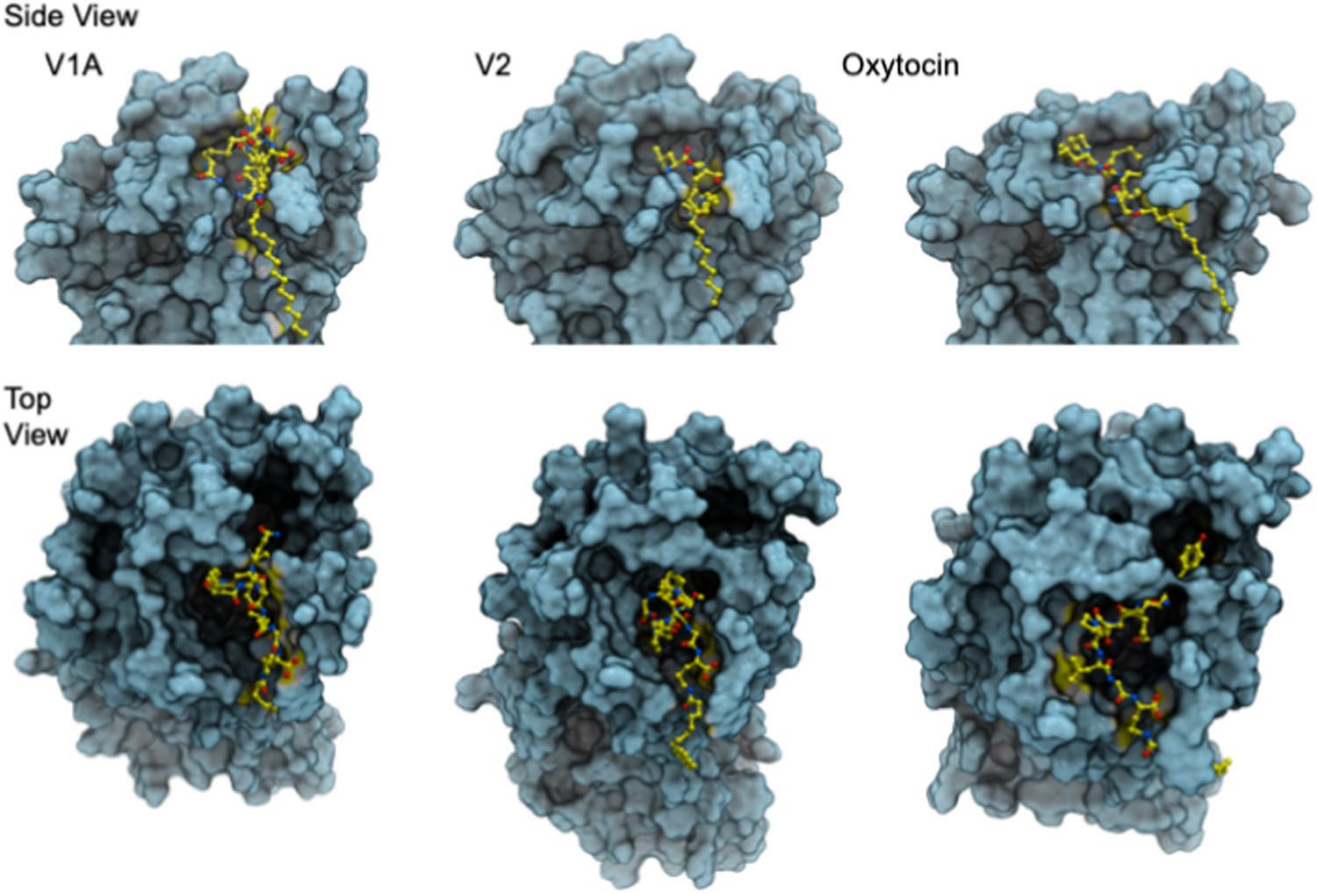
Top and Side view of the docked conformation of METx in V1a, V2 and OT receptors. METx is coloured yellow and the receptor is illustrated in a surface representation. The cyclic peptide head group of METx binds inside the receptor, while the hydrophobic acyl tail protrudes out of the receptor between a crevice formed between transmembrane helices 5 and 6. It is very likely that the hydrophobic tail anchors METx in the membrane, while the extent of interactions within the peptide binding site determines the depth of penetration. Further information on the simulations may be found in Supplementary Information Figs S2–S5.

To understand the METx binding to V1a, V2 and OT receptors, we carried out molecular modelling and *in silico* receptor binding studies (Supplementary Information Figs S2–S5 and Fig. 5). Since there are no crystal structures of these receptors available, homology models were built. The structure of human δ-opioid receptor (PDB id 4RWA) was identified as a template that exhibited a 30% sequence similarity with V1a receptor over 324 amino acids. The models of V2 receptor and OT receptor were constructed from V1a receptor. The overall sequence identity between V1a and V2 receptor is 46% over 309 residues and between V1a and OT receptor is 56% over 311 residues. In all cases, the models exhibited a characteristic conserved seven transmembrane G-protein coupled receptor (GPCR) fold motif. When structurally superimposed, the overall rmsd is>0.2 Å. The models were generated with the bifunctional peptide in place. This was done to ensure that the peptide-binding site is generated in all models. We then used the models to aid identification of residues contributing to the binding of METx. METx was docked in the binding sites using automated ligand docking. The METx molecule consists of a long hydrophobic acyl tail and a cyclic peptide head group. The cyclic peptide head group, rather than the entire METx was used in the docking studies, because the flexibility and hydrophobicity of the acyl chain of METx could impair the docking procedure. Analysing the METx head group-protein docking revealed several plausible docks in the binding site. From these docks, the final binding mode of the cyclic peptide was chosen on the basis in which the hydrophobic acyl tail could be added and that the extension of the tail did not alter the spatial position of the docked head group within the peptide-binding site. In each case the acyl tail extended to the outside of the receptor via a small crevice formed between transmembrane helices 5 and 6. The final model of METx and the receptor was then subjected to energy minimisation to relieve any steric clashes that might have arisen between the protein side chains and METx tail. See Supplementary Information Figs S2–S5.

A comparison of the docked METx is made between the V1a, V2 and OT receptors in Fig. 5. What is noteworthy is the extent to which the METx cyclic peptide head group penetrates into the receptor. The deepest and most interactions are observed between V2 receptor and METx, followed by OT receptor. Binding of METx in case of V1a receptor is shallow. This trend is also reflected in the binding score: V2 (−19.7)> OT (−13.4)> V1a (−9.9). It should be noted that the score is a unitless entity with no realistic meaning. However, it can be used as a relative measure of the binding affinity.

### METx Nanoparticle Formulations

METx was combined with Nanomerics Molecular Envelope Technology (N-palmiotyl-N,NN-trimethyl-N,N-dimethyl-N-monomethyl-6-O-glycolchitosan, also known as GCPQ^3,29^) to form METx loaded polymer nanoparticles of about 300–400 nm in size (Fig. 3b). The characteristics of the GCPQ^31^ were as follows: Mw = 7.12 kDa, Mn = 7.01, Mw/ Mn = 1.004, Mole% palmitoylation = 25%, Mole% quaternary ammonium groups = 12%. As can be seen the fibrous nature of METx was reduced by formulation with the polymer (Fig. 3a–c). As previously stated, nanofibres are known to form when lipidized peptides self-assemble in aqueous media^3,4,38^ and nanofiber length and nanofiber aggregation is reduced on coating with chitosan amphiphiles^3^ as the particles move from a nanofiber morphology to a more spherical morphology. This size reduction is also seen as the level of polymer in the formulation increases, with the large micron sized peak reducing with an increase in GCPQ content within the formulation (Fig. 3d). The large micron-size peak eventually becoming abolished at a METx, polymer ratio of 1: 10 (Fig. 3c,d). We assume that all nanofibers have been converted to nanospheres at aa METx, GCPQ mass ratio of 1: 10.

Over the 4 hour incubation the METx alone and METx, polymer formulations were completely degraded (Fig. 3e). The nanofiber morphology is known to stabilise peptides from degradation by the plasma *in vivo*^4^. However, METx nanofibres are completely degraded in the presence of 30% v/v rat plasma within 60 minutes (Fig. 3e). The order of stability was as follows: METx, Polymer (1: 5)> METx, Polymer (1: 7)> METx> METx, Polymer (1: 4). This demonstrates that the polymer nanoparticle stabilises the peptide from peptidase degradation, once a sufficient amount of polymer is included in the formulation [compare METx, Polymer (1: 4) to METx, Polymer (1: 5)], but once the particles become smaller, with increasing levels of GCPQ, they are vulnerable to peptidases [compare METx, Polymer (1: 5) to METx, Polymer (1: 7)]. We speculate that stabilisation against peptidase degradation, with the METx, Polymer (1: 5) formulation, has to originate from the fact that the peptide chains are protected from peptidase degradation by their hydrogen bonding with the polymer sugar molecules. The decrease in nanofiber morphology (length of the longest axis) indicates that inter-peptide hydrogen bonding to form the beta sheets has been reduced by the presence of GCPQ. It appears as if the inter-peptide interactions have been replaced by METx – GCPQ interactions. As GCPQ levels increase, the increased surface area of the smaller particles, seen with METx, polymer (1: 7) when compared to METx, polymer (1: 5) formulations, is likely to promote degradation by the plasma peptidases (Fig. 3e).

### *InVivo* Activity

**Figure 6:**
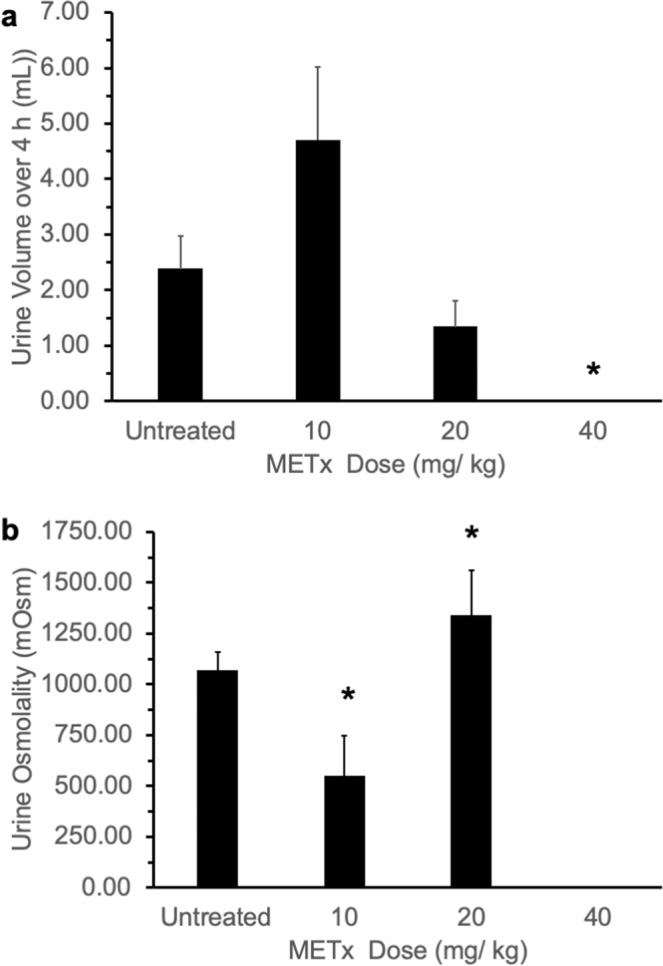
(**a**) Rat urinary output (n = 3, mean ± s.d.) and (**b**) rat urine osmolarity - following the intravenous administration of METx as a METx, polymer (1: 5 weight ratio) nanoparticle formulation, * = statistically significantly different from other doses.

In order to determine if agonism at the V2 receptor *in vivo* would result in a reduction of urine production, METx was studied *in vivo*. On measuring spontaneously voided urine volumes, it was observed that METx caused a dose correlated reduction in urine production (Fig. 6a), with the animals dosed with 40 mg kg^−1^ METx producing no urine at all over the 4 hour observation period. This reduced urine production is indicative of the *in vivo* agonist activity of METx at the V2 receptors in the kidney, causing water re-absorption and a reduced volume of urine (Figs. 1 and 5). It is noteworthy that despite the partial agonist activity of METx, there was a complete absence of measurable urine production with the highest dose of METx. The osmolality of the urine also increased significantly on administering METx at a dose of 20 mg kg^−1^ (Fig. 6b). This is expected as the urine volume decreases the level of solutes contained in a smaller volume would lead to a rise in osmolarity. It is noteworthy that lower doses of METx appeared to increase urine osmolarity with a similar trend towards increased volume observed (Fig. 6a,b). We are unsure if this observation is due to the antagonist activity of METx at lower doses (Fig. 4a), although this seems likely.

## Discussion

We have designed and synthesised an AVP analogue, which is essentially a self-assembling lipidized ten amino acid peptide (Fig. 2) which self assembles into nanofibers (Fig. 3a) in a similar manner to other lipidized peptides^3,4^. The lipidized peptide has a CMC of 18 μM that is similar to that seen with other lipidic peptides (8–14 amino acids long) with terminal palmitoyl chains (2–12 μM)^39^. Our goal was to explore if the lipidized peptide would have pharmacological activity despite its change in structure. In view of METx’ self-aggregation all METx receptor binding studies were carried out below the critical aggregation concentration of METx (<18 μM) to ensure that we were measuring the effects of the molecular monomer and not the nanofibres.

The lipidized peptide – METx - was a partial agonist at the V2 receptor (Table 2) and produced 60% of AVP’s activity (Fig. 4a). The positive control (AVP) validated these results with an EC50 of 0.93 nM in the MDCK cell line, in terms of cAMP production. This EC50 was similar to that seen with COS-1 cells expressing the V2 receptor (EC50 of AVP in the COS-1 cell line = 0.64 nM)^40^. However METx shows a more avid binding to the V2 receptor than AVP, reducing the magnitude of AVP’s effect by about 40% at high concentrations (Fig. 4a). METx works as a competitive antagonist to the activity of AVP and produces its own agonist activity as well. The strength of METx binding can be rationalised based on the depth of penetration of the METx cyclic peptide head group (Fig. 5). This enhances interactions with the receptor, while the hydrophobic tail anchors and stabilises METx in the lipid membrane via favourable interactions through a crevice formed between transmembrane helix 5 and 6.

**Table 2:** Activity of METx and AVP at the V2 and V1a receptors.

Compound	EC50 V2 receptor agonism (nM)	EC50 V1a receptor agonism (nM)	EC50 OT receptor antagonism (nM)
METx	2.7	> 1000	350^1^
AVP	0.93	13.1	Not determined

^1^antagonism against OT induced contraction frequency

METx is inactive at the V1a receptor up to a concentration of 1 μM (Fig. 4b). Desmopressin, a V2 receptor agonist^36^, is also inactive against the V1a receptor, while AVP is a V1a receptor agonist (Table 2, Fig. 4b). METx is active as a non-competitive antagonist at the OT receptor contained in uterine strips, inhibiting OT induced contractions at a concentration of 300 nM (100 fold greater than the EC50 of METx at the V2 receptor). From double reciprocal plots, METx was found to be a non-competitive inhibitor of OT (Table 1). *In silico* studies revealed the binding avidity to follow the trend V2 receptor> OT receptor> V1a receptor; with METx binding deep in the V2 receptor binding pocket (Fig. 5). These data point to the clear possibility that METx will act selectively at the V2 receptor *in vivo* (Fig. 1b). In order to test METx *in vivo*, we had to ensure that the peptide was not degraded rapidly within the plasma (Fig. 3e) and, as such, it was encapsulated within 300 nm GCPQ polymer^29,30,41^ particles (Fig. 3b). The formulation as GCPQ nanoparticles results in some protection from degradation providing an optimum amount of polymer is used. We have previously observed that encapsulation of lipidised enkephalin nanofibres within GCPQ reduced interactions of the nanoparticles with plasma proteins^3^ and encapsulation of the amphiphilic enkephalin and lipidized enkephalin within GCPQ nanoparticles reduced peptide degradation^2^. However, in the current study, unusually, having an excess of polymer made the peptide more susceptible to degradation (Fig. 3e), most possibly by increasing the surface area of the aggregates and exposing the non-beta sheet peptide chains to the plasma enzymes. Having established that encapsulation of METx within a particular level of polymer reduced plasma degradation (Fig. 3e), we then set out to examine the activity of METx *in vivo*. METx, when formulated as 300 nm polymer nanoparticles and injected intravenously, reduced the volume of spontaneously voided urine in a dose response manner (Fig. 6), indicating agonist activity at the V2 receptor *in vivo*. At the highest dose administered (40 mg kg^−1^, 7.2 μmoles to each rat) there was no urine produced by the rats during the 4 hour period and the reduction in urinary output was significant. The osmolality of the urine also increased at the intermediate dose (20 mg kg-1), further giving support to the *in vivo* agonist activity of METx at this particular dose.

These studies taken together demonstrate that the lipidic self-assembling peptide was pharmacologically active both *in vitro* and *in vivo* with selectivity for the V2 receptor. Previously we have prepared self-assembling nanofiber forming peptide amphiphiles using labile ester bonds to conjugate the lipid moiety to the peptide epitope and in essence formed prodrugs^3,4^, whereas in the current work the peptide was conjugated to the peptide epitope by a more stable amide bond (Fig. 2), thus presenting new possibilities to prepare self-assembling peptide drugs.

## Conclusions

We have produced a self-assembling AVP analogue with a lipidic side chain (METx). METx is a competitive agonist at the V2 receptor and more avid binder at the V2 receptor than AVP, with the lipid chain providing increased membrane anchoring. The lipid chain is implicated in the enhanced binding avidity for the V2 receptor. METx is inactive at the V1a receptor, indicating that vasoconstriction mediated side effects will not be a significant feature of METx. The compound shows antagonist activity at the OT receptor at a concentration that is 100 times in excess of the EC50 at the V2 receptor. On formulation into polymer nanoparticles and intravenous injection into healthy rats, METx shows dose correlated antidiuretic activity, indicating agonist activity at the V2 receptor *in vivo*.

## Supplementary Information

Supplementary Information.
