# Supplementary material for: A Self-Assembling Lipidic Peptide and Selective Partial V2 Receptor Agonist Inhibits Urine Production
Source: Sci Rep. 2020 Apr 29;10:7269. doi: 10.1038/s41598-020-64070-9 (PMC7190706; doi:10.1038/s41598-020-64070-9)
Supplement: Supplementary file 1 — Supplementary Information. [file 41598_2020_64070_MOESM1_ESM.pdf]

Supplementary Information - A Self Assembling Lipidic Peptide and Selective Partial V2 Receptor Agonist Inhibits Urine Production

Sunish Patel[1], Antonella Bavuso Volpe [1], Sahar Awwad[1], Andreas G. Schätzlein[1,2], Shozeb Haider[1], Liu Boqian[1], Ijeoma F. Uchegbu[1,2*]

1. UCL School of Pharmacy, 29-39 Brunswick Square, London, WC1N 1AX, UK
2. Nanomerics Ltd. 30-34 New Bridge Street, London EC4V 6BJ, UK

*corresponding author: Ijeoma.uchegbu@ucl.ac.uk

Materials and Methods

Receptor Binding Studies

V2 receptor

MDCK cells expressing the V2 receptor were cultured in Eagle's Essential Minimum Medium (EMEM) supplemented with foetal bovine serum (FBS, 10% v/v), L-Glutamine (1% v/v) and non-essential amino acids (NEAA 1% v/v). Cells were grown at 37°C in T-75cm^2 culture flasks under a humidified (95% air/ 5% CO_2) atmosphere. The cell culture medium was changed every 2 days. Cells were sub-cultured every 7 days or upon reaching confluence using trypsin /EDTA (0.25%w/v trypsin/ 0.01%w/v EDTA in phosphate buffered saline, 3 mL) solution to dissociate the cells and the cells reseeded at one third of their original density.

To determine the dose response curve, MDCK cells (passage number 10-20) were detached as described above using trypsin and seeded in transwell plates (12 mm transwell with 0.4μm pores) at a concentration of 500,000 cells per transwell and grown for 72 hours. The cell culture media was changed daily. On the day of the experiment, the cells were washed with Dulbecco's phosphate-buffered saline (DPBS) without calcium and magnesium and incubated for 1 hour in cell culture medium with isobutyl methylxanthine (IBMX, 0.5 mM). IBMX is a phosphodiesterase inhibitor, this prevents the breakdown of cAMP within the cells, allowing for total quantification of the analyte cAMP. After this, cells were incubated for 30 minutes with various concentrations of AVP or METx in cell culture medium with IBMX (0.5 mM). In order to reduce the concentration gradient of AVP or METx from the apical to the basolateral regions and vice versa, cells were treated from both the apical and basolateral chambers with the given concentration of AVP or METx dissolved in Dulbecco's phosphate buffered saline (DPBS) free of calcium or magnesium ions and with IBMX (0.5 mM, 0.5 mL in the transwell insert and 1.5 mL in the plate).

After 30 min incubation with the respective concentration of AVP or METx, the cells were lysed using the cAMP enzyme immunoassay (EIA) lysis buffer (diluted lysis reagent 1B, 0.5 mL, dodecyltrimethylammonium bromide 0.25% w/v, bovine serum albumin 0.02%w/v, preservative 0.01%w/v, acetate buffer 0.05M, pH = 5.8) and agitated for 10 minutes. cAMP was quantified from the cell lysate (0.1 mL) using a competition based EIA as per the protocol of the cAMP EIA as detailed below. This was carried out by preparing cAMP control standards in the lysis reagent 1B. The non-specific binding wells contained 0.1 mL of lysis reagent 1B and 0.1 mL of lysis reagent 2B (undisclosed contents). The blank wells contained 0.1 mL of lysis reagent 1B and the standard wells contained 0.1 mL of the standard cAMP solutions. The cell lysates (0.1 mL) were contained in the other wells. To each well, apart from the blank and non-specific binding wells, was added 0.1 mL of cAMP antiserum

(rabbit anti-cAMP). The plates were then covered, gently mixed and incubated at 3 – 5 °C for 2 hours. This was followed by the addition of the cAMP horseradish peroxidase conjugate (50 µL). The plate was then incubated again for 1 hour at 3 – 5 °C and the plate washed with wash buffer (phosphate buffer 0.01M, pH = 7.5, Tween 20 0.05% w/v, 0.4 mL). The enzyme substrate (3,3',5,5' -tetramethylbenzidine/ hydrogen peroxide, undisclosed concentration). was then added to the wells (0.15 mL) followed by the addition of sulphuric acid (0.1 M, 0.1 mL) and the plates read at 450 nm using an ELx808 microplate reader (Biotek instruments, Potton, UK). Each concentration of AVP or METx was tested in triplicate. The EC_{50} were calculated using Origin Pro 2016 (Origin Lab, USA). The concentration of cAMP per well was determined from equation 1.

$$\%B = \frac{standard\ or\ sample\ OD - NSB\ OD}{zero\ sample\ OD - NSB\ OD}\ X\ 100 \qquad 1$$

Where OD = optical density and NSB = non-specific binding.

A plot of % B vs the concentration of cAMP standards may then be used to calculate the concentration of cAMP in the samples.

All receptor binding assays were repeated three times.

Competition Studies

The same experimental set up as described above was followed for the seeding of MDCK cells and incubation with IBMX (0.5 mM) on the day of the experiment. For competition studies, the cells were incubated with a high concentration of AVP (10^{-7}M) and varied concentrations of METx (10^{-7} to 10^{-13}M). After 30 minutes of incubation with the respective concentration of AVP and METx, the cells were lysed using the cAMP EIA's lysis buffer for 10 minutes as per the cAMP EIA protocol outlined above. cAMP was quantified from the lysates (100µl) using a competition based EIA as per the protocol of the cAMP EIA described briefly above. The optical density was measured using at 450 nm using ELx808

microplate reader. Each concentration of AVP and METx was repeated in triplicate. The assay was repeated three times and the EC50 determined using Origin Pro.

V1a receptor

A7r5 cells were cultured in Dulbecco's Modified Eagle's Medium (DMEM) with high glucose concentrations (4.5 g/L glucose) and foetal bovine serum (FBS, 10% v/v). Cells were grown at 37°C and under a humidified 95% air /5% CO2 atmosphere in T75 cm^2 or T150 cm^2 culture flasks. The cell culture medium was changed every 2 to 3 days. When cells reached sub-confluence (80% to 90% coverage), they were subcultured using TrypLE™ Express (TrypLE™ Express is a replacement of the traditional trypsin reagent) and the cells reseeded at one third of their original density.

The IP1 ELISA was carried out using the IP-One ELISA kit. A7r5 cells (passage number: 20 - 23) were washed once in Dulbecco's Phosphate Buffered Saline (DPBS) and detached as described above using trypsin and then seeded in a 96 well plate at a concentration of 60,000 cells per well (in a 200 µL volume). The cells were incubated as described above for 22 hours. On the day of the experiment, the cell supernatant was then removed. The cells were then incubated with various concentrations of METx, AVP or desmopressin dissolved in the IP-One Stimulation Buffer (Hepes 10 mM, $CaCl_2$ 1 mM, $MgCl_2$ 0.5 mM, KCl 4.2 mM, NaCl 146 mM, Glucose 5.5 mM, LiCl 50 mM, pH = 7.4, 30 µL), with a set of control wells receiving only the IP-One Stimulation Buffer. After 1 hour of incubation with the respective concentrations of METx, AVP or desmopressin, the cells were then lysed using the IP-One Lysis Reagent (30 µL) by incubation as described above for a further 30 minutes. An aliquot of the cell supernatant (50 µL) was placed in the ELISA plate. The plate also contained a set of IP-ONE d-myo-inositol-1-phosphate (IP1) standards (50 µL) prepared according to the manufacturer's instructions. To the cell supernatant wells were added a solution of the IP-One d-myo-inositol-1-phosphate-horseradish peroxidase (IP1-HRP) conjugate (25 µL)

followed by a solution of the IP-One anti-IP1 monoclonal antibody (25 μL). Control wells examining non-specific binding contained only IP1-HRP (25 μL) and the IP-One diluent (75 μL). The plate was then incubated for 3 hours at room temperature while shaking. The wells were then washed three times with the IP-One wash solution (250 μL per well) followed by the addition of IP-One 3'3',5,5'-tetramethylbenzindine reagent (TMB, 100 μL, a HRP substrate, undisclosed concentration). In order to determine total enzymatic activity, to a control well was added TMB (5 μL). The wells were once again incubated for 25 minutes at room temperature in the dark. Finally, an IP-One Stop Solution (100 μL) was added to the well and the plates read at a wavelength of 450 nm and 620 nm. The Cells Net Optical Density (CNOD) was determined using Equation 2

$$CNOD = (COD_{450nm} - COD_{620nm}) - (NSOD_{450nm} - NSOD_{620nm}) \qquad 2$$

Where COD_{450nm} = the cell optical density at a wavelength of 450 nm, COD_{620nm} = the cell optical density at a wavelength of 620 nm, $NSOD_{450nm}$ = the non-specific binding well optical density at 450 nm, the $NSOD_{620nm}$ the non-specific binding well optical density at 620 nm. The percentage binding is determined using Equation 3.

$$\%B = \frac{CNOD}{CTNOD}\,100 \qquad 3$$

Where CTNOD = the control cells net optical density ($CTOD_{450nm} - CTOD_{620nm}$), with $CTOD_{450nm}$ and $CTOD_{620nm}$ being the optical density of control wells (originally containing only diluent) at wavelengths of 450 nm and 620 nm respectively.

The percentage V1a receptor activation (%S - % stimulation) is calculated as shown in Equation 4.

$$\%S = 100 - \%B \qquad 4$$

The assay was repeated three times.

OT Receptor

Figure S1

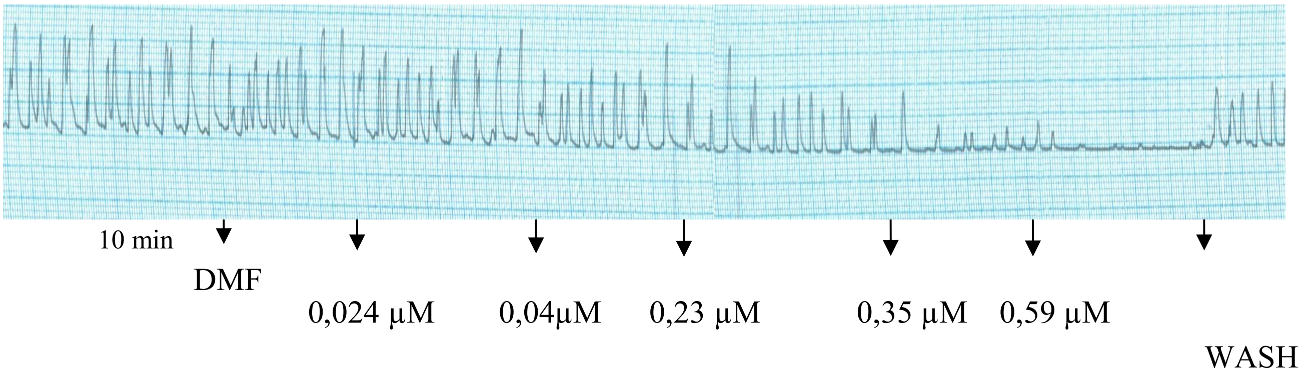

Figure S1 : The effect of METx on spontaneous uterine contractions – a tracing showing the effect of different doses of METx on spontaneous contractions.

Table 1: The addition of OT and METx to uterine strips to evaluate the effect of METx on uterine contractions

	OT induced contractions		Spontaneous contractions	
Time (minutes)	Compound added to uterine strips in 25 mL PSS	Final concentration	Compound added to uterine strips in 25 mL PSS	Concentration and volume added
0	None	Not applicable	None	Not applicable
60	OT	1.35 nM	dimethylformamide	100 µL
70	dimethylformamide	100 µL	METx	24 nM
80	METx	24 nM	METx	40.6 nM
90	METx	40.6 nM	METx	230 nM
100	METx	230 nM	METx	350 nM
110	METx	350 nM	METx	590 nM
120	METx	590 nM		

	OT induced contractions		Spontaneous contractions	
Time (minutes)	Compound added to uterine strips in 25 mL PSS	Final concentration	Compound added to uterine strips in 25 mL PSS	Concentration and volume added

Table 2: The addition of OT, METx and atosiban to uterine strips to evaluate the inhibition of OT induced contractions

Time (minutes)	Final Concentration of OT added to uterine strips in 25 mL PSS, final concentration	Final concentration of OT added to uterine strips before the addition of METx (100 nM), METx (300 nM), METx (600 nM) or atosiban (100 nM) added to uterine strips in 25 mL PSS
0	None	None
60	KCl 3.06 M, 0.5 mL	KCl 3.06 M, 0.5 mL
61	Wash twice	Wash twice
71	DMF 100 µL	METx (100 nM), METx (300 nM), METx (600 nM)
76	0.0405 nM	0.0405 nM
81	0.081 nM	0.081 nM
86	0.162 nM	0.162 nM
91	0.324 nM	0.324 nM
96	0.648nM	0.648 nM
101	1.3 nM	1.3 nM
106	2.7 nM	2.7 nM
111	5.4 nM	5.4 nM
116	10.8 nM	10.8 nM
121	21.6 nM	21.6 nM
126	43.2 nM	43.2 nM
131	86.4 nM	86.4 nM

Plasma stability - HPLC Analysis of METx

Table 3: HPLC Gradient for the analysis of METx

Time (minutes)	0	2	15	17	19	20	25
% acetonitrile	20	20	80	95	95	20	20

Results

In silico Receptor Binding

```
4RWA  41   RSASSLALAIAITALYSAVCAVGLLGNVLVMFGIVRYTKMKTATNIYIFNLALADALATS
V1AR  46   RNEELAKLEIAVLAV---IFVVAVLGNSSVLLALHRTPRKTSRMHLFIRHLSLAD-LAVA

4RWA  101  TLPFQSAKYLMETWPFG--ELLCKAVLSIDYYNMFTSIFTLTMMSVDRYIAVCHPVKALD
V1AR  102  FFQVLPQLCWDITYRFRGPDWLCRVVKHLQVFAMFASAYMLVVMTADRYIAVCHPLKTLQ

4RWA  159  FRTPAKAKLINICIWVLASGVGVP---IMVMAVTRPRDGAVV--CMLQFPSPSWYWDTVT
V1AR  162  -QPARRSRLMIATSWVLSFILSTPQYFIFSVIEIEVNNGTKTQDCWATFIQP---WGTRA

4RWA  214  KICVFLFA-FVVPILIITVCYGLMLL--------------------GSKEK---------
V1AR  218  YVTWMTSGVFVAPVVVLGTCYGFICYHIWRNIRGKTASSRHSKGDKGSGEAVGPFHKGLL

4RWA  253  -----------DRSLRRITRMVLVVVGAFVVCWAPIHIFVIVWTLVD-----IDRRDPLV
V1AR  278  VTPCVSSVKSISRAKIRTVKMTFVIVSAYILCWAPFFI-VQMWSVWDENFIWTDSENP--

4RWA  297  VAALHLCIALGYANSSLNPVLYAFLDEN-FKRCFRQL-C
V1AR  335  --SITITALLASLNSCCNPWIYMFFSGHLLQDCVQSFPC
```

```
V1AR_RAT  46   RNEELAKLEIAVLAVIFVVAVLGNSSVLLAL-HR-TPRKTSRMHLFIRHLSLADLAVAF
V2R_RAT   32   RDPLLVRAELALLSTIFVAVALSNGLVLGALIRRGRRGRWAPMHVFISHLCLADLAVAL
OXYR_RAT  34   RNEALARVEVAVLCLILFLALSGNACVLLAL--RTTRHKHSRLFFFMKHLSIADLVVAV

V1AR_RAT  103  FQVLPQLCWDITYRFRGPDWLCRVVKHLQVFAMFASAYMLVVMTADRYIAVCHPLKTLQQ
V2R_RAT   91   FQVLPQLAWDATDRFHGPDALCRAVKYLQMVGMYASSYMILAMTLDRHRAICRPMLAYRH
OXYR_RAT  91   FQVLPQLLWDITFRFYGPDLLCRLVKYLQVVGMFASTYLLLLMSLDRCLAICQPLRSLRR

V1AR_RAT  163  P--ARRSRLMIATSWVLSFILSTPQYFIFSVIEIEVNNGTKTQDCWATFIQPWGTRAYVT
V2R_RAT   151  GGGARWNR-PVLVAWAFSLLLSLPQLFIFAQRDV--GNGSGVFDCWARFAEPWGLRAYVT
OXYR_RAT  151  ----RTDRLAVLGTWLGCLVASAPQVHIFSLREV----ADGVFDCWAVFIQPWGPKAYVT

V1AR_RAT  221  WMTSGVFVAPVVVLGTCYGFICYHIWRNIRGKT-ASSRHSKGDKGSGEAVGPFHKGLLVT
V2R_RAT   208  WIALMVFVAPALGIAACQVL----IFREIHASLVPGPSERAGRRRRGRRTGSPSEG----
OXYR_RAT  203  WITLAVYIVPVIVLAACYGLISFKIWQNLRLKTAAAAAAAEGNDAAGGA------GRAAL

V1AR_RAT  280  PCVSSVKSISRAKIRTVKMTFVIVSAYILCWAPFFIVQMWSVWDENFIWTDSENPSITIT
V2R_RAT   260  ------AHVSAAMAKTVRMTLVIVIVYVLCWAPFFLVQLWAAWDPE---APLERPPFVLL
OXYR_RAT  257  ARVSSVKLISKAKIRTVKMTFIIVLAFIVCWTPFFFVQMWSVWDVN---APKEASAFIIA

V1AR_RAT  340  ALLASLNSCCNPWIYMFFSGHLLQDCVQSFPC
V2R_RAT   311  MLLASLNSCTNPWIYASFSSSVSSE-LRSLLC
OXYR_RAT  314  MLLASLNSCCNPWIYMLFTGHLFHELVQRFFC
```

Figure S2a (top): Sequence alignment of the template (PDB ID 4RWA) with V1a receptor.

Figure S2b (bottom): Sequence alignment between V1A, V2R and OT receptors across the modeled regions.

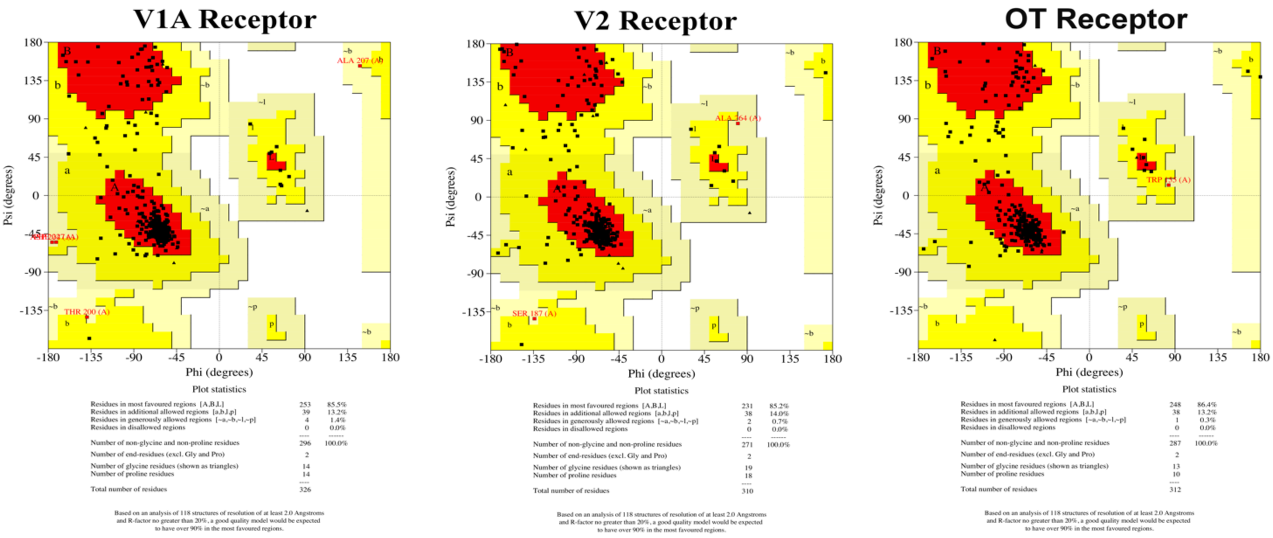

Figure S3: Stereochemical checking of the: V1, V2 and OT receptors via PROCHECK

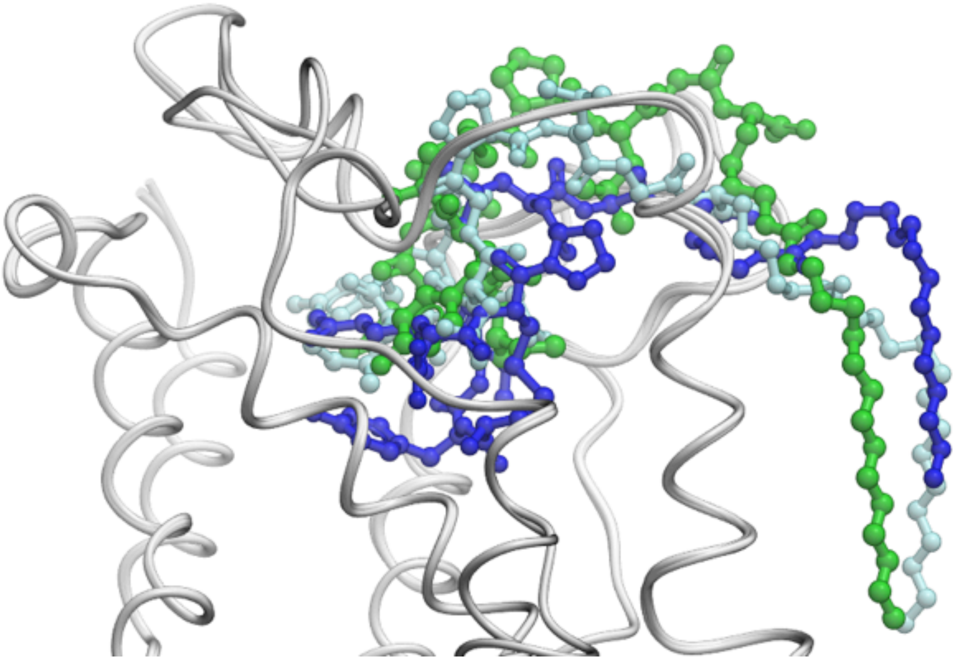

Figure S4: A comparison of the depth of penetration of METx in the receptors. METx penetrates the deepest in the V2 receptor (blue), followed by OT receptor (cyan) and then V1a receptor (green). The hydrocarbon tails anchor the cyclic peptide into the membrane.

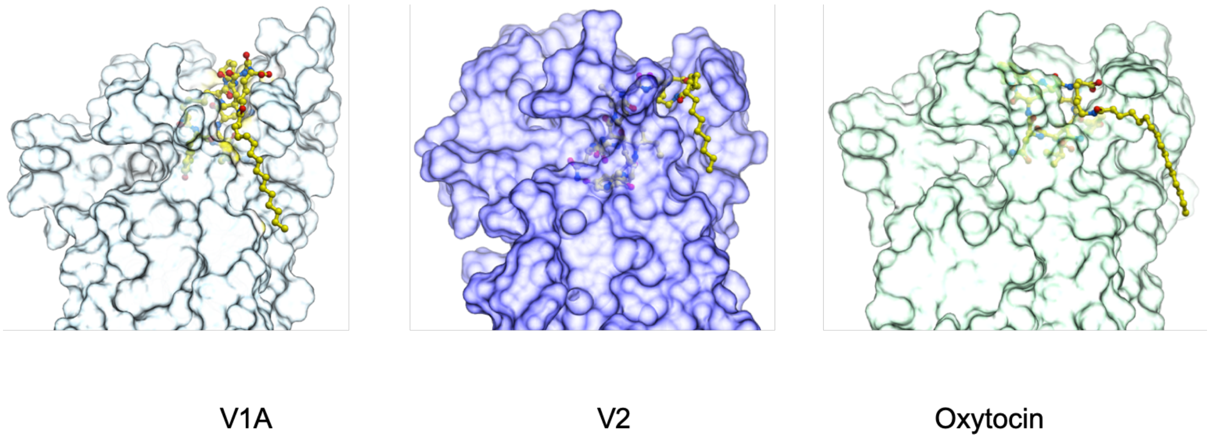

Figure S5: METx (yellow) docked into (a) V1A, (b) V2 and (3) Oxytocin receptors.
